# Extracellular histones are clinically relevant mediators in the pathogenesis of acute respiratory distress syndrome

**DOI:** 10.1186/s12931-017-0651-5

**Published:** 2017-09-02

**Authors:** Xin Lv, Tao Wen, Jiong Song, Dong Xie, Liang Wu, Xuemei Jiang, Ping Jiang, Zongmei Wen

**Affiliations:** 1 Department of Anesthesiology, Shanghai Pulmonary Hospital, Tongji University School of Medicine, Shanghai, 200433 People’s Republic of China; 2 Medical Research Center, Beijing Chao-Yang Hospital, Capital Medical University, Beijing, 100020 People’s Republic of China; 3 Department of Thoracic Surgery, Shanghai Pulmonary Hospital, Tongji University School of Medicine, Shanghai, 200433 People’s Republic of China; 4 Department of Emergency, Shanghai Pulmonary Hospital, Tongji University School of Medicine, Shanghai, 200433 People’s Republic of China

**Keywords:** Acute respiratory distress syndrome (ARDS), Extracellular histones, Cell damage, Systemic inflammation

## Abstract

**Background:**

Extracellular histones were recently identified as an inflammatory mediator involved in the pathogenesis of various organ injuries. This study aimed to examine extracellular histone levels and their clinical implications in acute respiratory distress syndrome (ARDS) patients and to explore histone-mediated effects through ex-vivo investigations.

**Methods:**

Extracellular histones, cytokine profiles and clinical data from 96 ARDS patients and 30 healthy volunteers were obtained. Human bronchial epithelial cells (BEAS-2B), human pulmonary artery endothelial cells (HPAEC), and human monocytic U937 cells were exposed to bronchoalveolar lavage fluid (BALF) collected from ARDS patients, and cellular damage and cytokine production were assessed. Furthermore, the effect of histone-targeted interventions by heparin or anti-histone antibody was evaluated.

**Results:**

Plasma and BALF extracellular histone levels were much higher in ARDS patients than in healthy controls. There was a significant association between extracellular histones and ARDS severity and mortality. In addition, extracellular histones correlated with an evident systemic inflammation detected in ARDS patients. Ex-vivo analysis further showed that ARDS patient’s BALF remarkably induced epithelial and endothelial cell damage and stimulated cytokine production in the supernatant of U937 cells. The adverse effects on these cells could be abrogated by heparin or anti-histone antibody.

**Conclusions:**

Extracellular histones in ARDS patients are excessively increased and may contribute to disease aggravation by inducing cellular damage and promoting systemic inflammation. Targeting extracellular histones may provide a promising approach for treating ARDS.

**Electronic supplementary material:**

The online version of this article (10.1186/s12931-017-0651-5) contains supplementary material, which is available to authorized users.

## Background

Acute respiratory distress syndrome (ARDS) is an acute inflammatory lung injury, associated with increased pulmonary vascular permeability, increased lung weight, and loss of aerated lung tissues [1, 2]. ARDS continues to have a high mortality rate (40%), despite advances in supportive care [3–5]. The reason why some cases progress to a worse condition while others progress toward resolution is not completely understood. Moreover, the pathophysiologic link between ARDS and the development of multiple organ dysfunction remains elusive. Therefore, the mechanisms related to the development of ARDS need to be explored.

The most common causes that precipitate ARDS can be a multitude of direct or indirect insults to the lung [6, 7]. It is noticeable that either direct or indirect insults can lead to a diffuse inflammatory response associated with accumulation of inflammatory cells, alveolar hemorrhage, and pulmonary edema, which subsequently results in a severe condition such as respiration failure [1, 3]. It is thereby suggested that systemic inflammation constitutes a common pathological feature during the development and progression of ARDS [8, 9]. Identifying the factors that trigger the systemic inflammation associated with ARDS may help to unravel its pathogenesis.

High levels of extracellular histones have recently been detected in many human diseases, and suggested as pivotal mediators of tissue injury and organ dysfunction [10, 11]. Existing evidence indicates that extracellular histones are cytotoxic to endothelial and epithelial cells, promote platelet aggregation and coagulation activation, and stimulate inflammatory responses acting through toll-like receptor (TLR) or inflammatory pathways [10, 12, 13]. In this study, we sought to examine whether extracellular histones are clinically involved with cellular damage and systemic inflammation implicated in the progression of ARDS, and whether extracellular histones could serve as a novel therapeutic target for ARDS.

## Methods

### Human subjects

This prospective observational study was approved by the Ethics Committee of Shanghai Pulmonary Hospital, Tongji University School of Medicine (Shanghai, P.R.China), which followed the recommendations of the Declaration of Helsinki for biomedical research involving human subjects. All subjects or their next of kin provided their informed consent for inclusion before they participated in the study. In total, 96 patients with ARDS were enrolled according to the Berlin definition of ARDS (acute hypoxemia, partial pressure of arterial oxygen (PaO_2_)/fraction of inspired oxygen (FiO_2_) ratio < 300 mmHg, and bilateral pulmonary infiltrates on chest radiography, and not explained by cardiac edema) [14]. Thirty healthy volunteers who undertook a routine physical examination were included as normal controls.

### Sample collection

The patients were studied within 24 h after ARDS diagnosis and monitored continuously during hospitalization. Arterial blood samples were obtained for blood gas analysis and patients were stratified for the degree of severity using the PaO_2_/FiO_2_ ratio as recommended. Mortality was defined as death occurring within 28 days after ARDS diagnosis. The baseline characteristics, demographic details, routine biochemical parameters, and clinical variables were recorded prospectively.

The peripheral blood samples and, when possible, bronchoalveolar lavage fluid (BALF) samples were collected from ARDS patients at admission (generally within 24 h after ARDS diagnosis). Plasma samples were separated, aliquoted and stored at −80°C. BALF samples were obtained by flushing the lungs with 20 ml saline, and centrifuged at 2000×g for 20 min at 4 °C and the supernatants were also stored at −80 °C for future analysis.

To assess the temporal changes of extracellular histones and their relationship to outcome, serial blood samples were obtained prospectively at defined time points in available ARDS patients. Survivors who required less than 7 days of respiratory support were classified as having a good evolution, and those who required more than 7 days of mechanical ventilation or who died were classified as having a poor evolution [15]. To those patients who died prior to days 7–9, they were removed from this dynamic observation.

### Measurement of extracellular histones and multiple cytokines

We first measured the levels of extracellular histones in the plasma and BALF of patients using a Cell Death Detection ELISA kit (Roche Applied Science, Germany). Purified mixed calf thymus histones were used to generate standard curves [12]. We then measured a panel of multiple cytokines (GM-CSF, IFN-γ, IL-1β, IL-2, IL-4, IL-5, IL-6, IL-9, IL-10, IL-12p70, IL-13, IL-17A, IL-18, IL-21, IL-23, IL-27, and TNF-α) using the ProcartaPlex™ Multiplex Immunoassay from Affymetrix eBioscience (San Diego, CA, USA), which permits the simultaneous measurement of various cytokines in a single sample [16].

### Quantification of LDH and MPO

Lactic dehydrogenase (LDH) is a cytoplasmic enzyme and its activity reflects the degree of tissue damage, whereas myeloperoxidase (MPO) represents an index of neutrophil, and monocyte/macrophage infiltration [17]. We measured the levels of LDH and MPO in the BALF of patients using commercial kits (Roche, Germany; BioVision, CA, USA), according to the manufacturer’s protocol.

### Ex-vivo experiments

The human bronchial epithelial cells (BEAS-2B), human pulmonary artery endothelial cells (HPAEC), and a human monocyte cell line (U937) were obtained from American Type Culture Collection (ATCC) and cultured in DMEM (Dulbecco’s Modified Eagle’s Medium, Sigma-Aldrich, St. Louis, MO, USA) supplemented with 10% fetal bovine serum (HyClone, Logan, UT, USA), 2 mM glutamine, and 100 U/ml penicillin/streptomycin (Sigma-Aldrich, St. Louis, MO, USA) in a 5% CO_2_ humidified atmosphere at 37 °C. After the cells grew to 80–90% confluence, they were incubated overnight with 50% of the BALF samples from ARDS patients, which were pooled from 26 ARDS patients. For interventional studies, heparin (250 U/ml, Sigma-Aldrich, St.Louis, MO, USA) or anti-histone H4 antibody (20 μg/ml) was added to the cultured cells.

Cell viability for BEAS-2B or HPAEC cells was determined using the Cell Counting Kit-8 (CCK-8, Sigma-Aldrich, St. Louis, MO, USA), according to the manufacturer’s instructions. LDH activities in cell culture supernatant were measured after cells were treated with the BALF. In addition, multiple cytokines in the supernatants of cultured U937 cells were analyzed using the ProcartaPlex™ Multiplex Immunoassay from Affymetrix eBioscience.

### Statistical analysis

For human data, values were expressed as medians and interquartile ranges or percentages unless otherwise stated. For cell culture data, values were expressed as the mean ± standard deviation (SD). Data were analyzed using an unpaired Student’s t test or Mann-Whitney test (for two groups), one-way analysis of variance (ANOVA) followed by Tukey post-tests (for more than two groups). Correlations between variables were assessed using Spearman’s rank correlation or Pearson correlation analysis. The results were considered statistically significant when *P* < 0.05. All statistical analyses were calculated using GraphPad Prism v5 (GraphPad Software, Inc., San Diego, CA).

## Results

### Extracellular histones are significantly elevated in the plasma of ARDS patients

The baseline demographic, clinical, and biochemical characteristics of the patients and the controls were shown in Table 1. Based on the Berlin definition, ARDS patients at admission were categorized into three groups according to degree of hypoxemia: mild (200 mmHg < PaO_2_/FiO_2_ ≤ 300 mmHg, *n* = 29), moderate (100 mmHg < PaO_2_/FiO_2_ ≤ 200 mmHg, *n* = 41), and severe (PaO_2_/FiO_2_ ≤ 100 mmHg, *n* = 26).

**Table 1:** Baseline demographical and clinical characteristics, and outcome of ARDS patients for sample analysis at enrollment

Characteristics	ARDS patients	Healthy controls	*p*-value
Sample Sizes	96	30	
Sex (Male/Female)	72/24	16/14	0.021
Age(years)	57.3 ± 14.3	41.1 ± 9.25	0.034
Etiology		NA	
Pneumonia	42		
Sepsis	27		
Aspiration	12		
Trauma	8		
Other factors	7		
Category			
Mild(200 mmHg < PaO2/FiO2 ≤ 300 mmHg)	29	NA	
Moderate(100 mmHg < PaO2/FiO ≤ 200 mmHg)	41	NA	
Severe(PaO2/FiO2 ≤ 100 mmHg)	26	NA	
28-day mortality, n(%)	24(25%)	NA	
SOFA score	11(8–15)	NA	

Data are expressed as median (interquartile range) or number of patients (%). Other factors include pancreatitis, transfusion, post-operation

We first measured the levels of extracellular histones in the plasma of these subjects and analyzed their correlation with disease severity and mortality. We observed that plasma histones were significantly increased in ARDS patients (median value, 41.5 μg/ml; range, 28.9–60.3) compared with healthy controls (median value, 0. 89 μg/ml; range, 0.37–1.16 μg/ml). Distinctively, extracellular histone levels were positively associated with the degree of ARDS severity. Severe ARDS patients had higher levels of extracellular histones (median value, 61.7 μg/ml; range, 57.3–70.9) when compared with moderate (median value, 40.2 μg/ml; range, 33.7–49.1) and mild patients (median value, 18.9 μg/ml; range, 15.2–33.6). There was also an inverse correlation between extracellular histones and PaO_2_/FiO_2_ values (*r* = 0.6716, *p* < 0.001; Additional file 1: Figure S1).

**Fig. 1:**
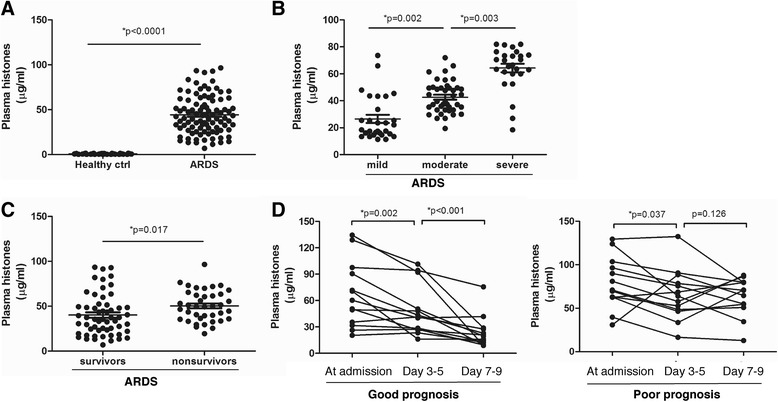
Elevated extracellular histones in the plasma of ARDS patients. **a**Median plasma histones were significantly increased in ARDS patients than in healthy controls (both *p* < 0.0001). **b** Plasma histone levels correlated with disease severity. Severe ARDS patients had higher extracellular histone levels than moderate or mild patients (all *p* < 0.05). **c** Median plasma histones were higher in nonsurvivor ARDS patients than those in survivors (*p* = 0.017). **d** Sequential plasma histone levels in ARDS patients at admission and days 3–5 and days 7–9. According to 28-day mortality, patients surviving the episode of ARDS (good prognosis) had a significant reduction in histone levels whereas nonsurvivors (poor prognosis) did not. Variables were expressed as median (interquartile range)

In addition, ARDS patients with elevated histone levels at admission had higher mortality; there was a significant difference in extracellular histone levels between nonsurviving patients and survivors (53.7 μg/ml [38.5, 63.9] vs. 38. 2 μg/ml [23.1, 49.6], *p* = 0.017). Notably, of these serial blood samples, the significant elevation of extracellular histones was observed at admission and peaked at days 3–5 with high levels still detectable at days 7–9. There was a remarkable difference in extracellular histone levels between patients with a good or poor evolution; the patients surviving the episode of ARDS showed a remarkable reduction in extracellular histones whereas nonsurvivors did not (Fig. 1). Taken together, these observations suggest that extracellular histones could reflect disease severity of ARDS and could predict prognosis in clinical situations.

There are various risk factors leading to the development of ARDS. We compared admission extracellular histones among ARDS patients with different etiologies and observed no significant difference (Additional file 2: Figure S2).

### Determination of extracellular histones in the BALF of ARDS patients

**Fig. 2:**
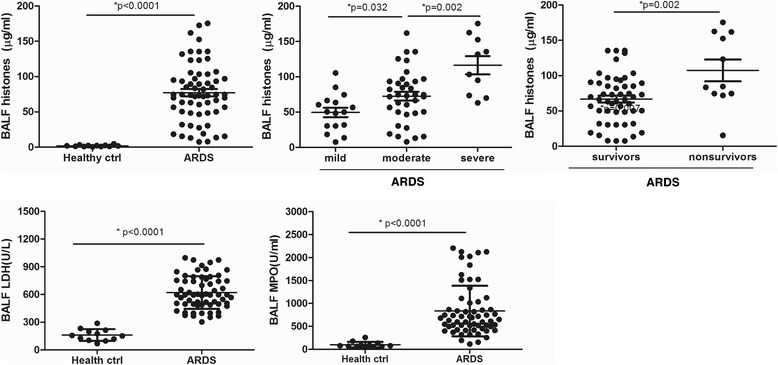
Extracellular histones were detected in the BALF of ARDS patients and their possible sources. Extracellular histones were also significantly higher in the BALF of ARDS patients than in healthy controls. Likewise, severe ARDS patients had higher BALF extracellular histone levels than moderate or mild patients, whereas non-survivor ARDS patients had higher BALF extracellular histone levels than survivors. Quantification of BALF LDH activity, a marker reflecting tissue damage, and BALF MPO activity, a marker of inflammatory cell activation, showed that both LDH and MPO levels were increased remarkably in ARDS patients compared to healthy controls, thus indicating a possible cellular sources for extracellular histones. Variables were expressed as median (interquartile range)

We further checked whether extracellular histones were present in the BALF samples of ARDS patients. The BALF measurements were available for 62 ARDS patients. As there were no BALF samples from healthy volunteers, we collected BALF samples to act as controls from 12 patients with lung benign nodules who were intubated for surgical reasons. Extracellular histone levels in the BALF of ARDS patients (median value, 74.8 μg/ml; range, 50.1–96.8) were higher than in the controls (median value, 1.85 μg/ml; range, 1.09–2.93). Similarly, BALF extracellular histones were associated with the degree of ARDS, and were significantly higher in non-survivor patients than in survivor patients (Fig. 2). It was thereby indicated extracellular histones are elevated uniformly in the BALF and peripheral blood of ARDS patients.

With respect to the source of extracellular histones, it has been suggested that extracellular histones can be passively released from severely injured tissues or actively released from the degradation of inflammatory leukocytes [11, 18]. Therefore, we assayed LDH activity, which reflects the degree of tissue damage, and MPO activity, which is indicative of inflammatory cell activation, in the BALF samples. The results showed that LDH and MPO were both markedly increased in the BALF of ARDS patients compared with the controls (Fig. 2). A simple linear correlation analysis showed a strong correlation between BALF histone levels and BALF LDH (*r* = 0.6161, *p* = 0.002) and between histones and BALF MPO (*r* = 0.5783, *p* = 0.004) (Table 2), which suggested that the elevated extracellular histones were possibly derived from damaged lung tissue cells as well as inflammatory cell infiltration during ARDS.

**Table 2:** Correlation of BALF histones with various variables in ARDS patients

	ARDS (*n* = 62)	
	r	P
LDH	0.6161	0.002*
MPO	0.5783	0.004*
IFN-γ	0.4876	0.021*
IL-6	0.7104	0.001*
IL-10	0.6158	0.002*
IL-12p70	0.5108	0.007*
IL-18	0.4012	0.043*
TNF-α	0.6368	0.002*

*p < 0.05 was considered to be statistically significant

**Fig. 3:**
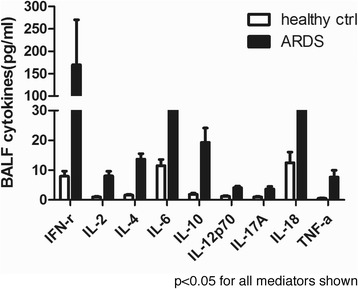
Detection of systemic inflammation in the BALF of ARDS patients at admission. Multiplex immunoassay for a panel of multiple cytokines was performed. Only 9 cytokines with significant differences (*p* < 0.05) between groups were shown. Variables were expressed as median (interquartile range)

It has been reported that patients with high levels of extracellular histones had enhanced inflammation [16, 19]. We therefore assessed the degree of systemic inflammation in ARDS patients at admission by measuring BALF cytokine profiles. We observed that there were 12 cytokines significantly increased in ARDS patients as compared with the controls (Fig. 3). Importantly, BALF histone levels positively correlated with BALF levels of IFN-γ, IL-6, IL-10, IL-12p70, IL-18 and TNF-α, all of which are indeed important markers of systemic inflammation (Table 2).

### Extracellular histones induce cell damage and promote cytokine production

**Fig. 4:**
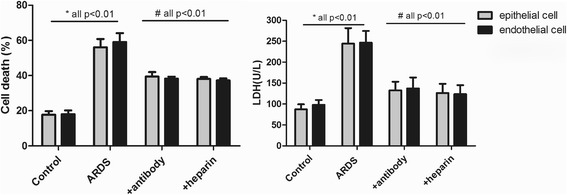
Induction of lung epithelial and endothelial cell damage by ARDS patient’s BALF. It showed that ARDS patients’ BALF could drastically induce human lung epithelial and endothelial cell damage after overnight incubation, whereas addition of anti-histone H4 antibody or heparin remarkably inhibited cell death caused by ARDS patient’s BALF. Likewise, LDH levels were significantly increased in supernatant of these cells after overnight incubation of ARDS patient’s BALF, whereas addition of anti-histone H4 antibody or heparin could reduce LDH levels. Variables were expressed as mean ± standard deviation (SD)

**Fig. 5:**
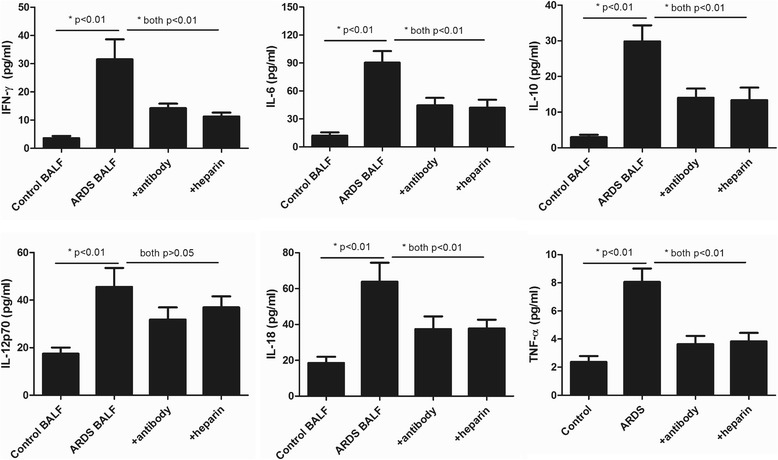
Stimulatory effects of ARDS patient’s BALF on human monocytic cells. It showed that 6 histone-related cytokines were all notably increased in the supernatant of ARDS BALF-treated human monocytic U937 cells, whereas addition of anti-histone H4 antibody or heparin could decrease these cytokine levels (all *p* < 0.05). Variables were expressed as mean ± standard deviation (SD)

Bronchial epithelial cell or endothelial cell integrity and function are essential for normal gas exchange, and injury to these cells may lead to the progression of ARDS [15, 20]. To determine whether extracellular histones in the BALF of patients accounted for lung injury in patients, we first investigated the direct effects of BALF extracellular histones on human bronchial epithelial cells (as the major cells responsible for gas exchange in the lung) as well as on endothelial cells, and then we assessed whether BALF histones adversely affected human monocytes. The results showed ARDS patient’s BALF that contained high levels of histones was cytotoxic to BEAS-2B or HPAEC cells, as evidenced by significantly increased cell death and elevated LDH levels (Fig. 4). The rate of cell death was equivalent in both cell populations, independent of the BALF used. In addition, overnight culture of human U937 monocytes with ARDS patient’s BALF led to a remarkable elevation of histone-related cytokines (IFN-γ, IL-6, IL-10, IL-12p70, IL-18 and TNF-α) in the supernatants of cell culture (Fig. 5). By contrast, the BALF from the controls had little effect on these cells.

Previous studies have shown that anti-histone treatment (e.g., anti-histone H4 antibody, APC, pentraxin 3 (PTX3) or heparin) was significantly protective in various inflammatory conditions [21–23]. We investigated in this study whether heparin, which can bind histones, had some protective effects for ARDS. Anti-histone H4 antibody was also included as positive control. We showed that administration of heparin or anti-histone antibody markedly inhibited ARDS patient’s BALF-induced epithelial and endothelial cell damage or activation of U937 cells (Figs. 4 and 5), which was indicated by decreased cell death rate in two groups (BEAS-2B and HPAEC cells) as well as by decreased histone-related cytokine levels in the supernatants of cultured U937 cells. These findings thus suggest a direct relationship between extracellular histones and cellular damage and systemic inflammation observed in ARDS, and provide a possible novel strategy for clinical intervention of ARDS.

## Discussion

ARDS is commonly seen in critically ill patients, generally resulting from lung-specific or multiple systemic insults [3]. So far, the pathogenesis of ARDS is not completely understood, but intense systemic inflammation is obviously involved [6, 8]. Following primary injuries (infection, chemical, trauma) to the lungs (alveolar epithelium, lung endothelium), the epithelial/endothelial cell border is disrupted and promotes the recruitment and activation of inflammatory cells into interstitial and bronchoalveolar spaces. The overwhelming inflammatory response not only attacks the lung but also causes extrapulmonary organ dysfunction and ultimately high mortality [6, 8]. However, the mechanisms related to activation of systemic inflammation during ARDS are absent.

Extracellular histones have recently been discovered as a new class of highly tissue-damaging mediators [10, 24]. Normally, histones are located within the nucleus and have essential roles in chromatin remodeling and gene transcription; however, histones will play distinct roles when they are released into extracellular milieu. Accumulating evidence shows that extracellular histones can lead to systemic inflammatory and toxic responses in a single or a combined manner [22, 25]. High levels of extracellular histones are directly cytotoxic to endothelial and epithelial cells, as well as several other cell types, possibly through disrupting cellular membrane and leading to increased transmembrane conductance, calcium influx, cell swelling and cytolysis [26–28]. In addition, extracellular histones can regulate coagulation and thrombosis by promoting platelet aggregation and impairing the protein C-thrombomodulin system [29]. Most importantly, extracellular histones can act as damage-associated molecular pattern (DAMP) molecules to trigger an inflammatory response [13, 24, 30].

In this study, we aimed to investigate the roles of extracellular histones in the context of ARDS with a hypothesis that extracellular histones may serve as a disease biomarker or as a potential therapeutic target for ARDS. Here, we observed a marked increase of extracellular histones in ARDS patients compared with the controls. Of note, high levels of extracellular histones were found to be associated with disease severity and mortality of ARDS, indicating that extracellular histones may serve as good predictors for disease progression and outcome. In addition, we confirmed the presence of extracellular histones in the BALF and plasma of ARDS patients; we speculated that extracellular histones may be released first into the pulmonary alveoli and then transported via the blood flow to other organs. As to the source of extracellular histones in BALF, our work and the prior literature suggested that this was possibly from massive dead/dying lung cells, from inflammatory cell infiltration, or both [31, 32]. However, there may be other cell sources of histones in the lung that require further investigation.

Systemic inflammation is generally considered a major cause of lung injury in the development of ARDS [9]. Many studies have suggested that extracellular histones may trigger an inflammatory response through TLR or inflammasome pathways [13, 24]. Our work showed that a broad spectrum of canonical inflammatory and chemokines were remarkably increased in the BALF and associated with extracellular histone levels, thereby supporting the idea that extracellular histones may be the main culprit of intense systemic inflammation during ARDS. Ward and colleagues have described that extracellular histones are essential effectors of inflammation in experimental ARDS [31]. Another group has also linked extracellular histones with trauma-associated ARDS [32]. Our work expands on previous studies to assess whether extracellular histones have direct cell damaging and inflammation promoting effects, and whether extracellular histones can potentially be utilized for therapeutic purposes with ex-vivo investigations. We demonstrated that ARDS patient’s BALF that contained high levels of extracellular histones significantly decreased human lung epithelial/endothelial cell integrity and stimulated human monocytic cells to produce massive cytokines. A specific anti-histone neutralizing antibody could prevent these adverse effects, thus confirming a cause-effect relationship between extracellular histones and cellular damage and systemic inflammation. It is of important translational relevance that the blockade of extracellular histones may be a promising therapeutic strategy for ARDS. Therefore, we checked the interventional effects of other histone-blocking agents such as heparin in this study. It has been reported that heparin, which can bind histones, showed some protective effects in histone-mediated inflammatory injuries including sepsis, and acute liver injury [16, 23]. Here, we observed that heparin had a cytoprotective effect similar to that of anti-histone antibody in inhibiting extracellular histone-induced cytotoxicity or cytokine production, which may provide a potential approach for the clinical management of ARDS.

**Fig. 6:**
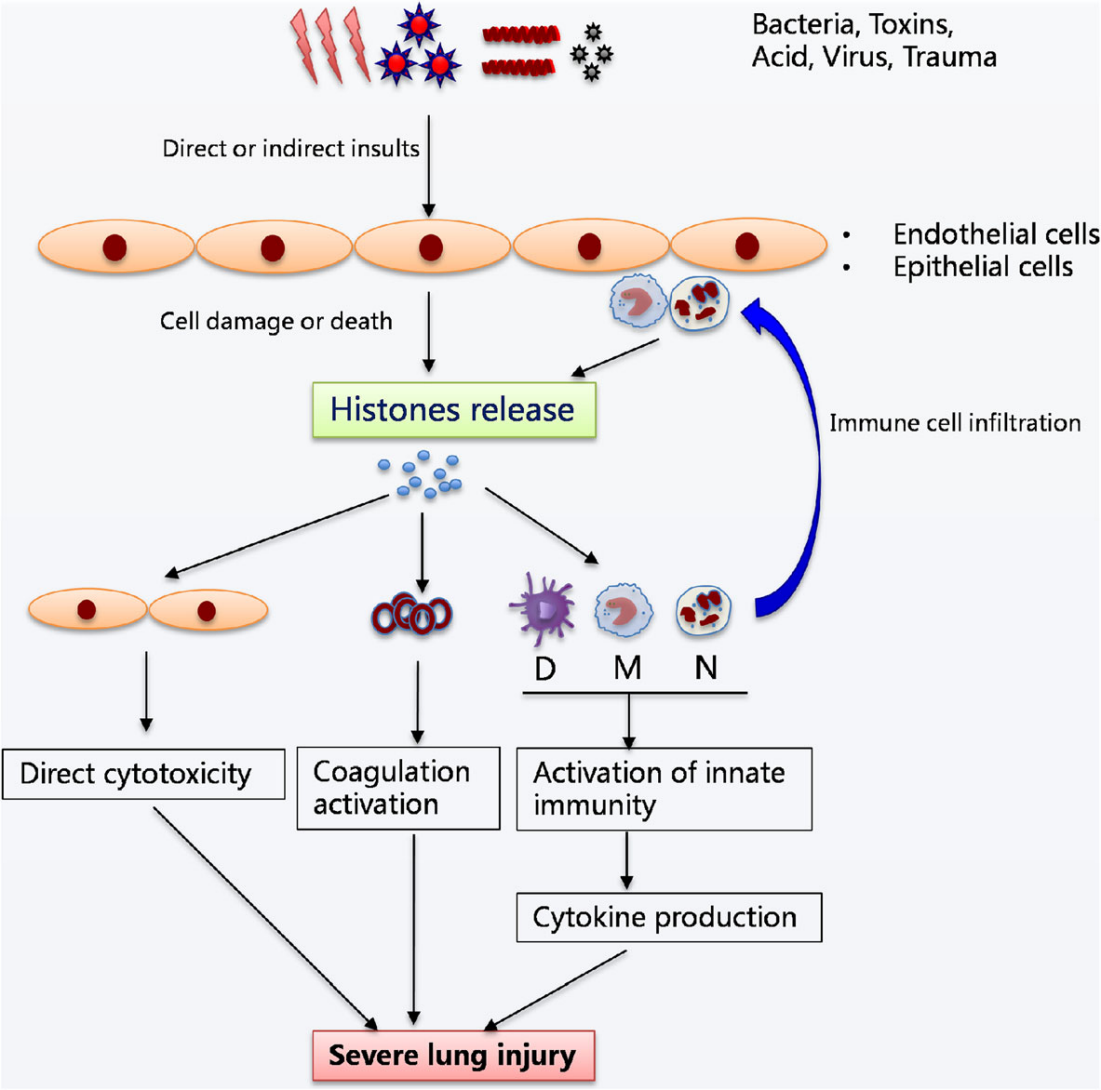
The hypothesized mechanistic model of extracellular histones in mediating cellular injury and systemic inflammation associated with ARDS. It is proposed that various insults (lung specific or extrapulmonary) cause extensive death of lung endothelial and/or epithelial cells or induce inflammatory cell infiltration, which subsequently leads to massive release of histones into extracellular spaces. The presence of high levels of extracellular histones may exert multiple biological effects including direct cytotoxicity, platelet aggregation, and activation of systemic inflammation by promoting cytokine production, which in turn attracts more inflammatory cells and enhance inflammation that eventually contribute to the pathogenesis of ARDS

In addition, we showed that extracellular histone levels are not related to different causes of ARDS, implying that extracellular histones may be independent of the underlying etiology of ARDS, and histone-mediated cellular injury and inflammation may be a common pathway during the progression of ARDS. Based on these findings, we conclude that either direct lung injuries or systemic insults may lead to extensive cell death and result in the massive release of histones into extracellular spaces. The detrimental effect of excessive histone release is particularly important to lung disease, because histones can expand more easily in the pulmonary alveoli, causing direct cellular damage [33]. Extracellular histones may also be transported via the blood flow to distant organs, further promoting extrapulmonary organ dysfunction and even death through their distinct effects (e.g., histone-enhanced inflammation, coagulation, and cytotoxicity). There is an uncontrolled positive feedback loop in which the damaged cells or activated immune cells release histones, which causes more damage, adds histone release, and contributes to the disease progression. We therefore propose a hypothetical model of extracellular histones in mediating cellular injury and systemic inflammation in ARDS (Fig. 6).

There are certain limitations in this study. First, this was only an observational, case-controlled study that was prone to selection biases. Indeed, healthy controls were no well age, and gender-matched, although these factors were not potential confounders (data not shown). Second, despite no significant differences being observed in the histone levels among patients with different etiologies, due to limited number of ARDS patients, it would be more cautious to analyze whether extracellular histones are related to etiologies or not, which requires further investigation. Another limitation is the lack of various pathological controls for this study such as sepsis, trauma or pneumonia patients without ARDS. In addition, unavailability of BALF samples from normal controls is also a limitation. To ensure that the data were comparable, we attempted to use BALF from patients with benign lung nodules, in which no abundant extracellular histones were detected.

## Conclusions

Extracellular histones play a pathological and targetable role in the development and progression of ARDS. Determining extracellular histone levels and understanding their biological effects are likely translatable to improving the clinical management of ARDS. More importantly, targeting extracellular histones by heparin or anti-histone antibody provides a promising therapeutic strategy. More histone blocking agents should be explored in the future.

## Additional files

Additional file 1: Figure S1. A correlation between extracellular histones and PaO_2_/FiO_2_ values. (TIFF 51 kb)

Additional file 2: Figure S2. Extracellular histones among ARDS patients with different etiologies. (TIFF 58 kb)

## Abbreviations

ALI: Acute lung injury
APC: Activated protein C
ARDS: Acute respiratory distress syndrome
BALF: Bronchial alveolar lavage fluid
DAMP: Damage-associated molecular pattern
LDH: Lactic dehydrogenase
MPO: Myeloperoxidase
