# Supplementary figures and images for: Extracellular histones are clinically relevant mediators in the pathogenesis of acute respiratory distress syndrome
Source: Respir Res. 2017 Sep 2;18:165. doi: 10.1186/s12931-017-0651-5 (PMC5581408; doi:10.1186/s12931-017-0651-5)

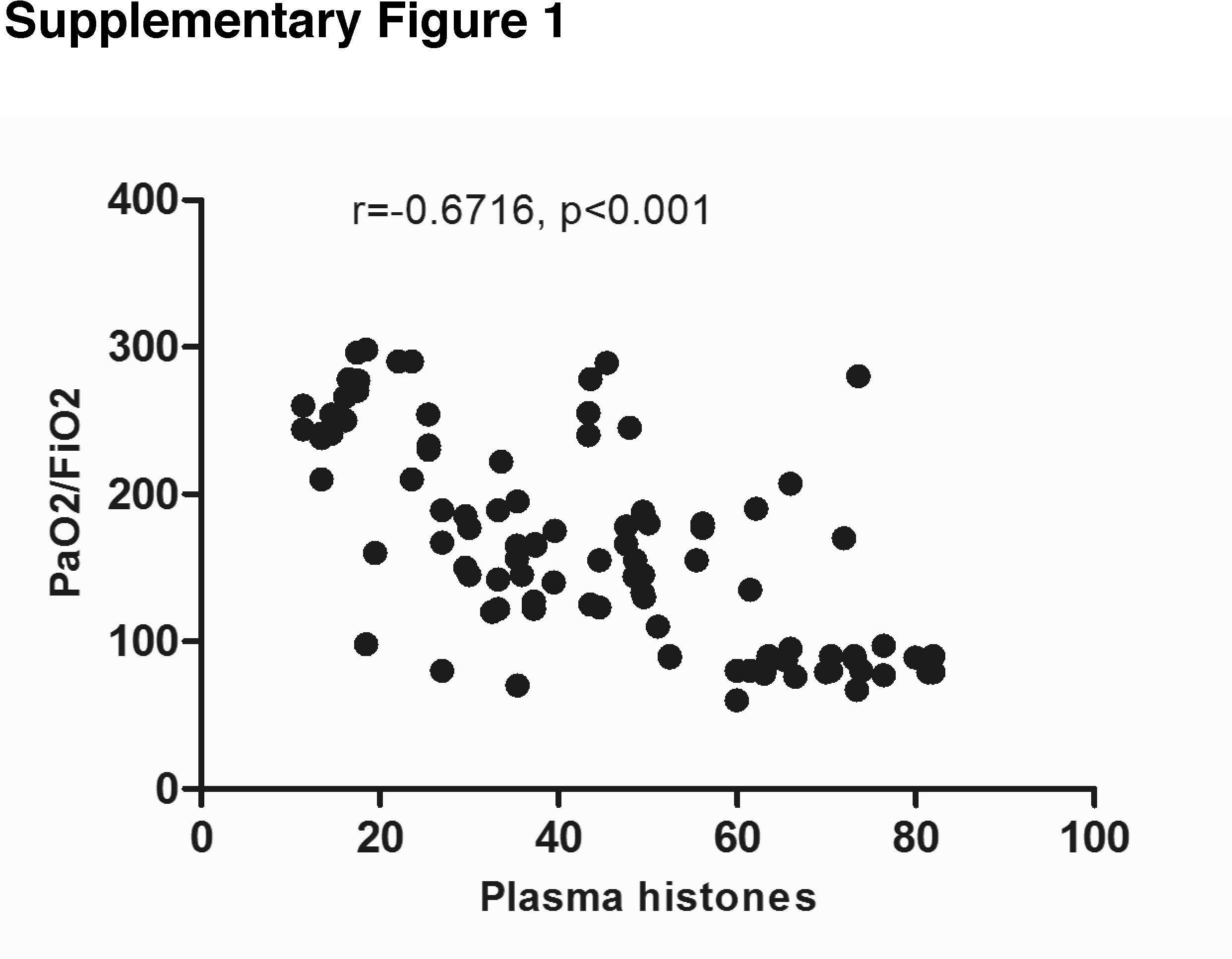

Supplement: Supplementary file 1 — A correlation between extracellular histones and PaO2/FiO2 values. (TIFF 51 kb) [file 12931_2017_651_MOESM1_ESM.tif]

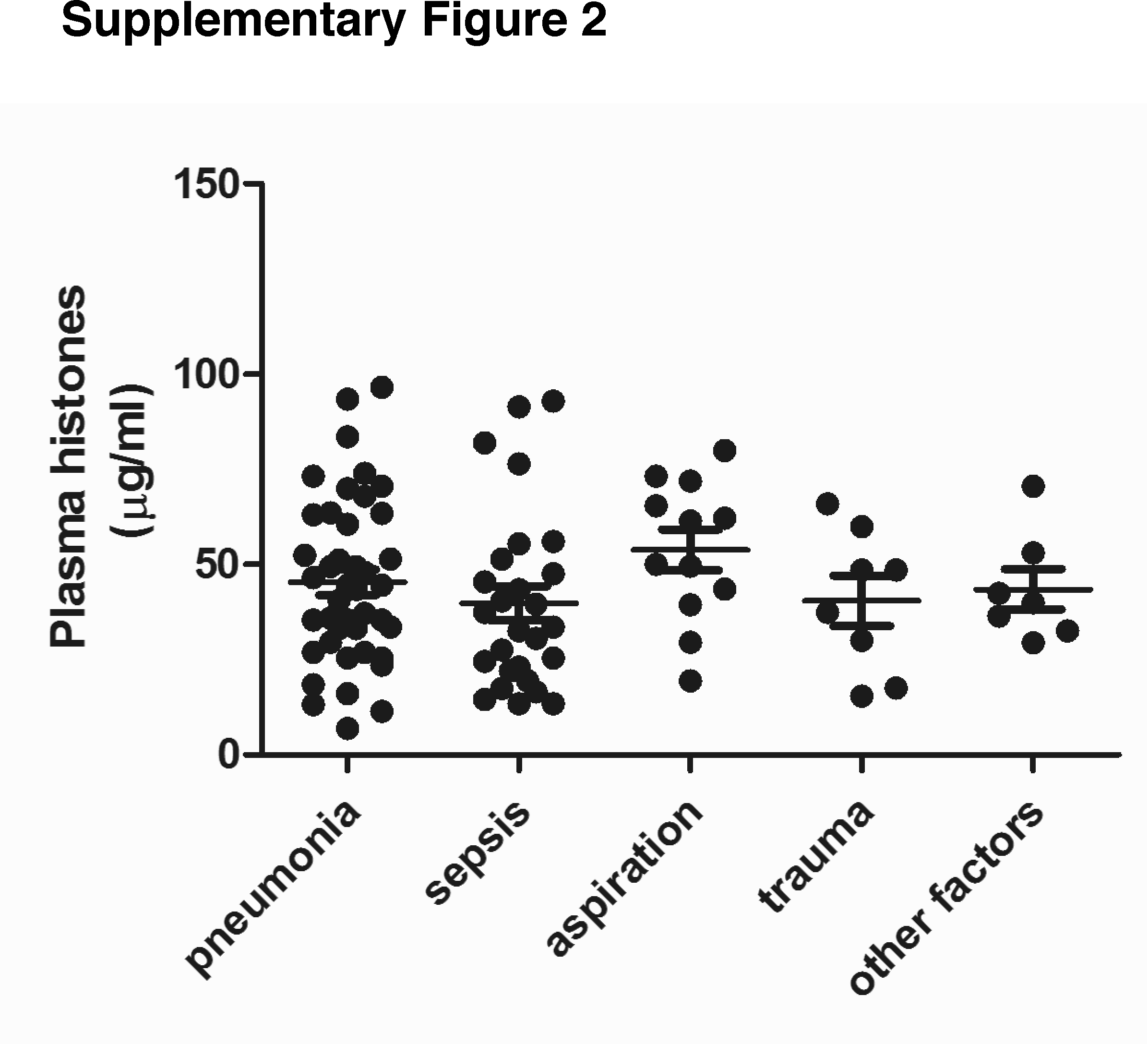

Supplement: Supplementary file 2 — Extracellular histones among ARDS patients with different etiologies. (TIFF 58 kb) [file 12931_2017_651_MOESM2_ESM.tif]
